# Morphology-guided attention networks for explainable skin cancer detection under clinical uncertainty

**DOI:** 10.3389/fonc.2026.1804329

**Published:** 2026-04-07

**Authors:** Muhammad Zaheer Sajid, Muhammad Fareed Hamid, Zepa Yang, Mohammad Alhefdi, Shrooq Alsenan, Yunyoung Nam

**Affiliations:** 1 Department of Electrical and Computer Engineering, George Mason University, Fairfax, VA, United States; 2 Department of Computer Software Engineering, National University of Sciences and Technology, Military College of Signals (MCS), Islamabad, Pakistan; 3 Department of Computer Science and Engineering, Soonchunhyang University, Asan, Republic of Korea; 4 Computer Engineering Department, King Khalid University, Abha, Saudi Arabia; 5 Information Systems Department, College of Computer and Information Sciences, Princess Nourah bint Abdulrahman University, Riyadh, Saudi Arabia

**Keywords:** clinical decision support, deep learning, dermoscopic image analysis, explainable artificial intelligence, lesion segmentation, morphology-guided attention, skin cancer detection, uncertainty-aware learning

## Abstract

Accurate and reliable skin cancer detection from dermoscopic images remains challenging due to large visual variability, overlapping lesion appearances, and inherent clinical uncertainty. To address these issues, this work proposes a morphology-guided attention framework for explainable and uncertainty-aware skin lesion classification. The system integrates lesion segmentation to preserve clinically meaningful morphological structures, followed by an attention-based classification network that emphasizes diagnostically relevant regions while suppressing background artifacts. Visual attention and attribution maps are generated to provide transparent explanations aligned with established dermoscopic criteria. In addition, an uncertainty estimation module is incorporated to quantify prediction confidence and identify ambiguous or out-of-distribution cases for safe clinical triage. The proposed approach is evaluated on publicly available dermoscopic datasets and achieves classification accuracy 99.12% with a recall rate above 99% for malignant lesions, demonstrating strong sensitivity for early cancer detection. Experimental results show that morphology-guided attention improves both classification performance and interpretability compared to conventional deep learning models. Furthermore, uncertainty-aware predictions enhance model reliability by reducing overconfident errors in challenging cases. These findings indicate that the proposed framework offers a robust, explainable, and clinically relevant solution for automated skin cancer screening under real-world conditions.

## 1 Introduction

Skin cancer is one of the most common diseases in the world and its cases are increasing day by day. This disease also creates a big medical and financial burden. According to global cancer reports, a large number of melanoma and non-melanoma skin cancer cases are recorded every year. Because of this reason, fast and scalable screening systems are very important for early diagnosis (1). In the United States, cancer statistics also show a high number of melanoma cases and deaths every year. This shows that early detection and proper risk analysis are very necessary in clinical practice (2). Dermoscopy helps doctors to see the inner structure of skin lesions, but correct diagnosis is still difficult. This is because benign and malignant lesions look similar in many cases, and malignant lesions can also change in appearance for different patients, skin colors, devices, and image conditions.

Recent advances in deep learning have improved the automatic skin lesion recognition system. A well-known research work showed dermatologist-level classification performance by using deep neural networks trained on large image datasets (3). Many community efforts, such as the International Skin Imaging Collaboration (ISIC) challenges, provided standard tasks and evaluation methods which helped in faster development of different techniques and fair comparison of results (4, 5). Public dermoscopy datasets like HAM10000 and PH^2^ also supported supervised learning by providing large and diverse skin lesion images with proper imaging protocols (6, 7). Furthermore, the Derm7pt dataset introduced structured clinical labels based on checklist criteria, which helped in multi-task learning and clinically meaningful model training (8).

Despite many improvements in skin cancer detection systems, there are still two main problems which limit safe use in real clinical environment. First, many high accuracy models are difficult to understand and they do not provide clear clinical explanations. In real dermatology practice, doctors depend on lesion morphology such as border irregularity, pigment network, streaks, globules, regression structures, and abnormal vascular patterns. Therefore, the explanation results must be related to these visible dermoscopic features instead of only using general saliency maps. Second, clinical deployment is strongly affected by uncertainty. Some images are unclear, some lesions are rare, and some images are different due to device changes, lighting conditions, or data noise. Also, previous studies reported that many public datasets contain duplicate images and other data problems which can affect performance results if not handled carefully (9). For this reason, a reliable system should not only give high accuracy, but also provide confidence scores and proper rejection or referral for uncertain cases. Many ensemble-based and transformer-based models have shown good performance on benchmark datasets, but they still need proper uncertainty modeling and clinically meaningful explanation methods for real-world use (10, 11). Segmentation-based and attention-based approaches have also shown that focusing on lesion regions can improve both robustness and interpretability of the system (12, 13).

**Figure 1:**
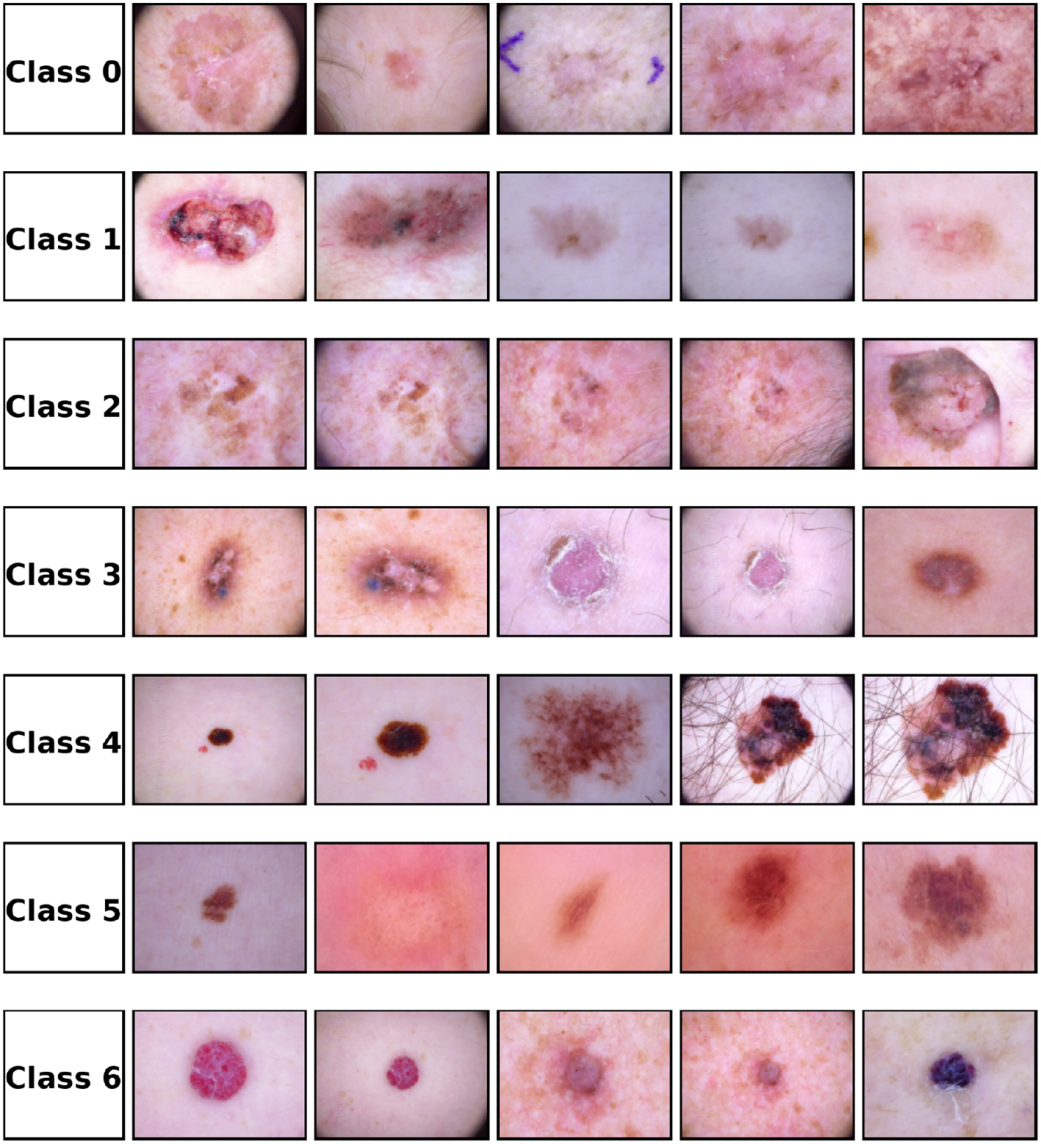
Representative dermoscopic images of the seven skin lesion classes from the HAM10000 dataset. Class 0: NV (Melanocytic nevi), Class 1: MEL (Melanoma), Class 2: BKL (Benign keratosis-like lesions), Class 3: BCC (Basal cell carcinoma), Class 4: AKIEC (Actinic keratoses), Class 5: VASC (Vascular lesions), and Class 6: DF (Dermatofibroma). Each row corresponds to one lesion class, and the columns show different samples of the same category. Black borders are included to clearly separate individual images and illustrate variations in lesion morphology, color, and texture across different diagnostic classes.

The proposed work presents a morphology-guided attention framework for automatic skin cancer detection and classification. This system is designed to be explainable and uncertainty-aware. First, the lesion area is localized using a segmentation-guided module. This helps to preserve important morphological features such as lesion boundaries, asymmetry patterns, and internal texture changes. These morphology-based features are then passed to an attention-based classification network. This network focuses on important lesion regions and reduces the effect of background areas. For better interpretation, visual attention and attribution maps are generated. These maps highlight the lesion regions that mostly affect the final prediction and follow standard dermoscopic criteria. An uncertainty estimation mechanism is also added to measure the confidence of the prediction. This helps to identify unclear or out-of-distribution cases. Low-confidence samples are sent for expert review for safe clinical usage. Overall, this framework provides accurate skin lesion classification with transparency, clinical importance, and reliable performance in real-world conditions. The visual diversity of dermoscopic lesion images for different disease classes is shown in **Figure 1**.

### 1.1 Research contributions

This work proposes a clinically aligned, explainable, and uncertainty-aware deep learning framework for automated skin cancer detection from dermoscopic images. The main contributions of this research are summarized as follows:

- The segmentation guided preprocessing module is introduced to preserve the clinically meaningful lesion morphology such as lesion boundaries, asymmetry and internal texture patterns. This module helps the classification network to focus on the diagnostically relevant lesion regions instead of focusing on background artifacts.
- The framework is integrate the spatial and channel attention mechanisms to enhance the discriminative feature learning. This attention guided representation is improve the sensitivity for the subtle visual cues like irregular borders and heterogeneous pigmentation which are strongly associated with the malignant lesions.
- Clinically interpretable attention and attribution maps are generated to show the lesion regions which contribute more in the final prediction. These explanations are aligned with the segmentation masks and provide transparent and morphology-consistent visual evidence for clinical validation.
- The uncertainty estimation module is added in the system using Monte Carlo dropout method. This module is used to measure the prediction confidence of the model. If the uncertainty is high then that case is marked for expert review. This helps for safe clinical decision and also reduce the risk of wrong prediction in unclear cases.
- The extensive experiments are performed on HAM10000 dataset. The proposed framework achieve more than 99% classification accuracy with high recall for malignant lesion. The ablation study, explainability analysis and uncertainty calibration experiments are also conducted. The results confirm that the system is effective, interpretable and reliable.
- By jointly addressing performance, interpretability, and uncertainty estimation, the proposed framework provides a robust and clinically trustworthy solution for automated skin cancer screening under real-world conditions.

## 2 Related work

### 2.1 Datasets and benchmarking for dermoscopic analysis

Public datasets and community challenges are very important for the development of automated skin lesion analysis. The ISIC challenge series provided standard tasks like classification and segmentation. It also provided common evaluation protocols and large participation. Because of this, different methods can be compared in a systematic way (4, 5). HAM10000 is a widely used multi-source dermoscopy dataset. It contains common pigmented skin lesion images and it is mainly used for multi-class classification tasks (6). PH^2^ is another well-known dataset. It is frequently used for lesion segmentation and validation studies (7). Derm7pt introduced structured labels based on the seven-point checklist. This dataset supports multi-task learning and clinically meaningful analysis (8). However, the quality of datasets and the selected evaluation protocols can affect the reported performance. Some studies showed that the ISIC dataset contains duplicate images and other irregular issues. Because of this, careful data curation and robust evaluation are required (9).

### 2.2 CNN-based classification and multi-scale learning

CNN backbones are trained on large skin lesion datasets and these are used in many classification systems. In early work, the researchers showed dermatologist-level performance in skin cancer classification using deep neural networks and large image datasets (3). After that, many challenge-based pipelines used strong CNN backbones, data augmentation, and ensemble learning to reduce class imbalance and improve the system performance. For example, multi-resolution EfficientNet ensembles with metadata branches gave strong results in ISIC challenge tasks. This shows that multi-scale feature extraction and system-level optimization are helpful for better classification performance (10). However, only high accuracy is not enough to provide correct explanations and reliable confidence under clinical uncertainty. Recent deep learning architectures have continued to improve classification performance through optimized network design and training strategies. For example, SkinNet-14 (25) proposed an optimized convolutional neural network architecture specifically designed for skin lesion classification, achieving improved accuracy with reduced computational cost. Similarly, large-scale benchmark datasets such as BCN20000 (26) provide more diverse and clinically realistic dermoscopic images, highlighting the importance of robust and generalizable classification systems that can operate under real-world imaging variability. These recent developments demonstrate that modern CNN-based systems can achieve strong diagnostic performance; however, many of these approaches primarily focus on classification accuracy and do not explicitly integrate morphology-guided feature alignment, explainable visualization, and uncertainty-aware decision support within a unified framework.

### 2.3 Morphology-guided learning via segmentation and attention

Because the dermatologic diagnosis is mainly based on morphology, many systems use lesion segmentation or region-based methods so that the classification is focused on lesion boundaries and internal structures instead of background noise. U-Net is a basic and commonly used architecture for biomedical image segmentation and it is mostly used for lesion localization (12). Attention-based segmentation networks also improve the boundary detection and reduce the effect of artifacts in dermoscopic images. For example, ASCU-Net uses attention gates with spatial and channel attention to enhance the lesion segmentation performance (13). Morphology guidance can be applied by using lesion masks or extra morphology tasks to guide the attention toward clinically important regions, which helps to generate explanations that are more suitable with clinical understanding. More recent segmentation approaches have further improved lesion localization using attention-based feature refinement. For example, SkinAttn-Net (29) introduces multi-level attention mechanisms within a U-Net framework to enhance lesion boundary delineation and improve structural feature preservation. These advances demonstrate the importance of attention-guided segmentation for accurate morphology extraction. However, most existing segmentation-based methods focus primarily on improving segmentation accuracy and do not directly integrate morphology guidance into downstream classification with uncertainty-aware explainability, which is essential for clinically reliable diagnostic systems.

### 2.4 Transformer and attention-based recognition

Transformer style attention is used in skin lesion recognition to capture long range dependencies and global context. Transformer based models and hybrid CNN–Transformer designs give good results on standard benchmark datasets. This shows that global attention is helpful to detect subtle morphological patterns which are spread in different lesion regions (11). However, attention is not equal to explanation and transformer models can still be over confident. Therefore, attention mechanism should be combined with morphology guidance and uncertainty estimation to achieve clinically trustworthy results. Recent transformer-based and hybrid architectures have further improved skin lesion recognition by combining global attention modeling with multi-task learning. For example, vision transformer-based diagnostic systems (27) have demonstrated improved performance in joint segmentation and classification tasks by capturing long-range spatial dependencies. These approaches highlight the effectiveness of transformer-based attention in modeling complex lesion patterns. However, transformer attention alone does not guarantee clinically meaningful explanation or reliable uncertainty estimation. Without morphology-guided alignment and uncertainty modeling, these systems may still produce overconfident predictions and explanations that are not fully consistent with clinical diagnostic criteria.

### 2.5 Explainability: attribution and visual evidence

Explainability methods are widely used to show model evidence and to build clinical trust. Grad-CAM method is used to localize important regions by using gradients on convolutional feature maps and generate heatmaps which are overlaid on the input image (14). Integrated Gradients method works by calculating gradients from a baseline image to the original input image and provides attribution values with theoretical properties for better interpretability (15). However, explanation methods can give misleading results if the model is uncertain or if the explanation is not truly representing the decision process. Therefore, morphology-guided and uncertainty-aware explainability is needed for more reliable and trustworthy clinical interpretation.

### 2.6 Recent deep learning frameworks for automated skin cancer diagnosis

Recent deep learning frameworks such as SkinDeepNet (30) have proposed automated pipelines for early skin cancer diagnosis using multi-stage feature extraction and classification strategies. These systems demonstrate improved performance through architectural optimization and automated feature learning. More recently, dermoscopy-informed deep learning models have further improved diagnostic accuracy by incorporating lesion-specific preprocessing and convolutional feature learning tailored to dermatological imaging characteristics. For example (31), developed a dermoscopically informed deep learning framework for classification of actinic keratosis and squamous cell carcinoma, demonstrating that domain-specific preprocessing and CNN-based architectures can significantly enhance diagnostic reliability in clinical dermoscopy analysis.

In addition, hybrid deep learning and radiomics-based diagnostic systems have shown promising results in improving classification accuracy and generalization. Wang et al. (2024) proposed a hybrid deep learning and radiomics framework that integrates dermoscopic image features with clinical metadata to improve diagnostic performance across diverse lesion types, highlighting the importance of combining deep learning representations with clinically relevant feature modeling (32).

These recent developments demonstrate that modern deep learning systems are capable of achieving high diagnostic performance through architectural optimization, hybrid feature integration, and domain-specific modeling. However, most existing frameworks primarily focus on improving classification accuracy and do not explicitly address the combined challenges of morphology-guided attention, explainable visual alignment with lesion morphology, and uncertainty-aware clinical decision support. In contrast, the proposed framework integrates segmentation-guided morphology preservation, attention-based feature refinement, explainable visualization aligned with lesion structure, and uncertainty-aware prediction within a unified architecture, which improves both diagnostic reliability and clinical interpretability.

### 2.7 Clinical uncertainty: Bayesian approximations, ensembles, and calibration

Uncertainty-aware deep learning is used to identify when the model is not confident in the prediction. This helps in safe decision making and proper case referral. MC Dropout is a technique where dropout is applied during testing time and it provides uncertainty estimation using Bayesian approximation (16). Deep ensemble method uses multiple models to estimate uncertainty and it also improves accuracy and calibration performance (17). Many calibration studies show that deep learning models are not always well calibrated, so calibration evaluation and correction methods are required (18). Evidential deep learning uses a Dirichlet distribution to model prediction evidence and estimate uncertainty. This method allows the system to avoid making predictions when the evidence is not sufficient (19). By combining uncertainty modeling with morphology-guided attention, the system can provide more reliable explanations and support safer clinical decisions for unclear cases. The summary of related work on skin lesion analysis, including datasets, segmentation methods, explainability techniques, and uncertainty models, is shown in **Table 1**.

**Table 1:** Representative prior work in skin lesion analysis.

Ref.	Direction	Core idea	Explain.	Uncert.
(3)	Large-scale CNN classification	End-to-end deep learning for expert-level skin cancer recognition	No	No
(4) (5),	ISIC benchmarking	Standardized challenges and evaluation protocols for lesion analysis	No	No
(6)	Dataset (HAM10000)	Large multi-source dermoscopy dataset for pigmented lesions	N/A	N/A
(7)	Dataset (PH^2^)	Curated dermoscopy dataset for benchmarking	N/A	N/A
(8)	Clinically structured learning	Seven-point checklist-aligned multi-task learning	Yes	No
(9)	Benchmark integrity	Analysis of duplicates and evaluation risks in ISIC datasets	Indirect	Highlights risk
(10)	Ensemble multi-resolution	Multi-scale EfficientNet ensembles with metadata	Indirect	Partial
(12)	Segmentation backbone	U-Net for biomedical lesion localization	Yes	No
(13)	Attention-based segmentation	Attention gates with spatial/channel attention	Yes	No
(11)	Transformer recognition	Transformer-based lesion classification	No	No
(14)	Attribution visualization	Grad-CAM class-discriminative heatmaps	Yes	No
(15)	Attribution (axiomatic)	Integrated Gradients for principled explanations	Yes	No
(16)	Bayesian approximation	MC Dropout for predictive uncertainty	No	Yes
(17)	Predictive uncertainty	Deep ensembles for scalable uncertainty estimation	No	Yes
(18)	Calibration analysis	Miscalibration analysis of modern neural networks	No	Yes
(19)	Evidential learning	Dirichlet-evidence uncertainty modeling	No	Yes

## 3 Methodology

**Figure 2:**
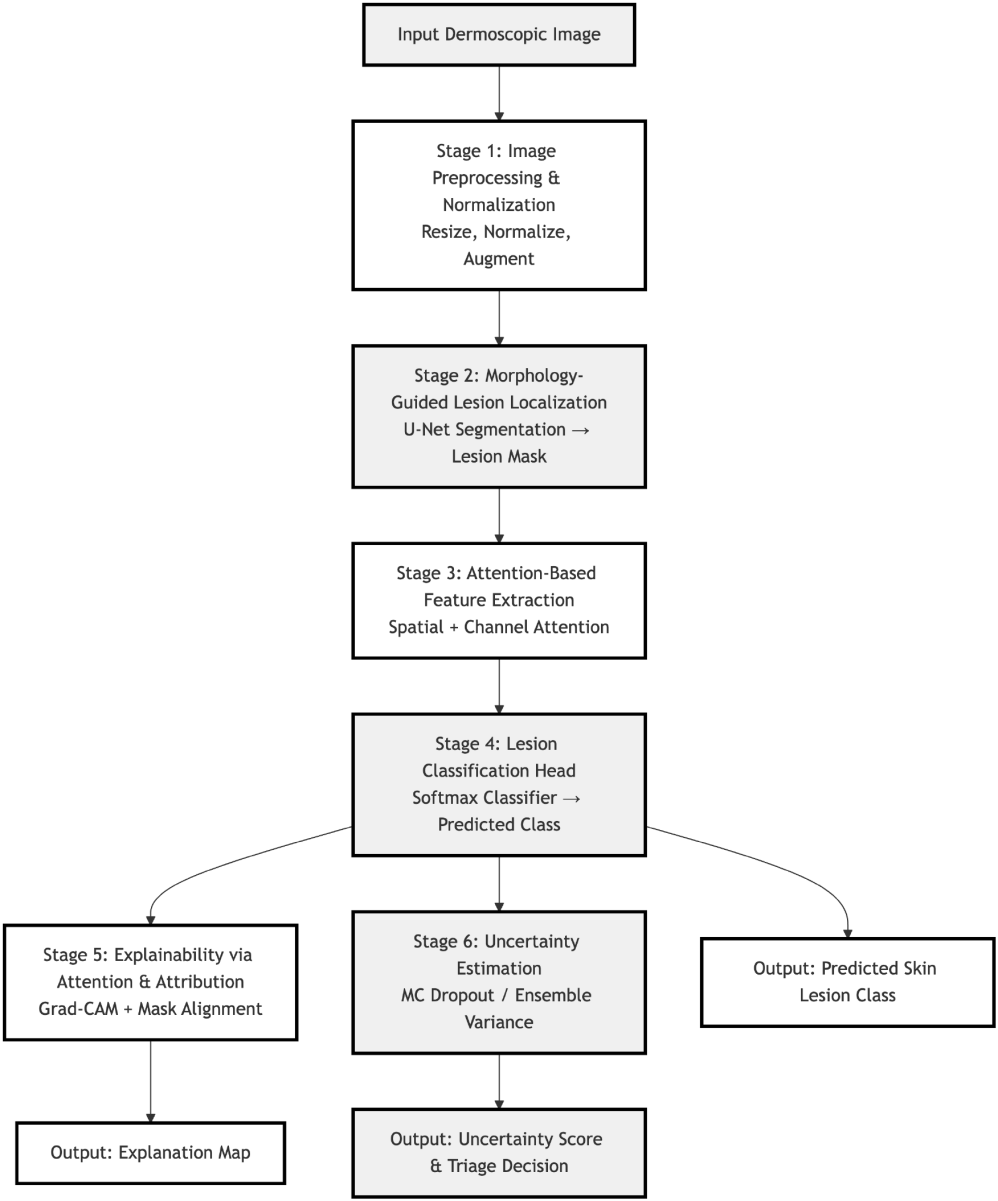
Workflow of the proposed morphology-guided attention network for skin cancer detection.

The proposed framework is an explainable and uncertainty-aware deep learning system for automatic skin cancer detection and classification using dermoscopic images. The complete workflow of this system is shown in **Figure 2**. This figure explain all main processing steps, starting from input image preprocessing and lesion localization to morphology-guided attention-based classification and uncertainty estimation. The framework is design to focus on clinically important morphological structures and also provide clear visual explanations with reliable confidence results for safe clinical decision support.

All input dermoscopic images are first resize into fixed resolution of 224×224 pixels and normalized using ImageNet statistics. This standardization help to ensure stable training and consistent feature extraction for different datasets and imaging devices. To improve the system performance in real-world conditions such as illumination changes, camera noise, hair artifacts, and color variation, different data augmentation techniques are applied during training. The augmentation include random rotation, horizontal and vertical flipping, brightness and contrast adjustment, and color jittering. These preprocessing steps help the model to generalize better for unseen clinical images.

The first stage of the framework is based on lesion localization using a segmentation guided module. In this stage, a U-Net based architecture is used to generate accurate lesion masks. These masks help to preserve important morphological information such as lesion boundaries, asymmetry, and internal texture patterns. The generated masks are then used to guide the feature extraction process by removing background regions and highlighting the important lesion areas. This morphology guided localization helps the classification network to focus only on clinically meaningful structures instead of background information, as shown in Fig.??.

In the second stage, the extracted morphology aware features are passed to an attention based classification network. This network uses both spatial attention and channel attention mechanisms to highlight important lesion regions and important feature channels. This attention guided learning helps the model to capture fine visual patterns such as irregular borders, different pigmentation, and abnormal texture structures which are commonly found in malignant lesions. By using morphology based attention, the proposed framework improves the classification accuracy and also provides better interpretability.

To provide transparent and clinically interpreable results, visual explanation maps are generated for each test image. Gradient-based methods and attention visualization techniques are used to highlight the lesion regions which are most important for the final classification decision. These explanation maps are matched with the segmented lesion areas so that doctors can check whether the model is focusing on correct morphological features such as lesion edges, color irregularities, and structural asymmetry. This explainable design helps to increase trust in the automated prediction system and also supports clinical validation.

In addition to classification and explainability, the proposed framework also includes an uncertainty estimation module to measure the prediction confidence. During the testing phase, multiple forward passes are performed using dropout-based Bayesian approximation or ensemble-based methods. The prediction variation is then used to calculate epistemic uncertainty for each input image. The cases with high uncertainty are marked for expert review, which helps in safe diagnosis and reduces the risk of incorrect predictions in difficult or unclear cases. This uncertainty-aware mechanism improves the clinical reliability of the system, as shown in **Figure 2**.

The overall training objective is combination of segmentation loss, classification loss, attention regularization loss, and uncertainty-aware calibration loss. The segmentation loss is used to ensure accurate lesion localization. The classification loss is used to improve the diagnostic performance of the system. The attention regularization loss helps the network to focus on meaningful morphological regions. The calibration loss is used to improve the confidence reliability of the predictions. This multi-objective optimization strategy helps the framework to achieve high diagnostic accuracy with better interpretability and robustness under real-world clinical uncertainty.

Overall, the proposed methodology integrates morphology-guided learning, attention-based feature modeling, explainable visualization, and uncertainty-aware decision support into a single unified pipeline. As shown in **Figure 2**, this end-to-end workflow is designed to provide accurate, transparent, and clinically trustworthy skin cancer predictions for real-world deployment.

## 4 Architecture of the proposed framework

**Figure 3:**
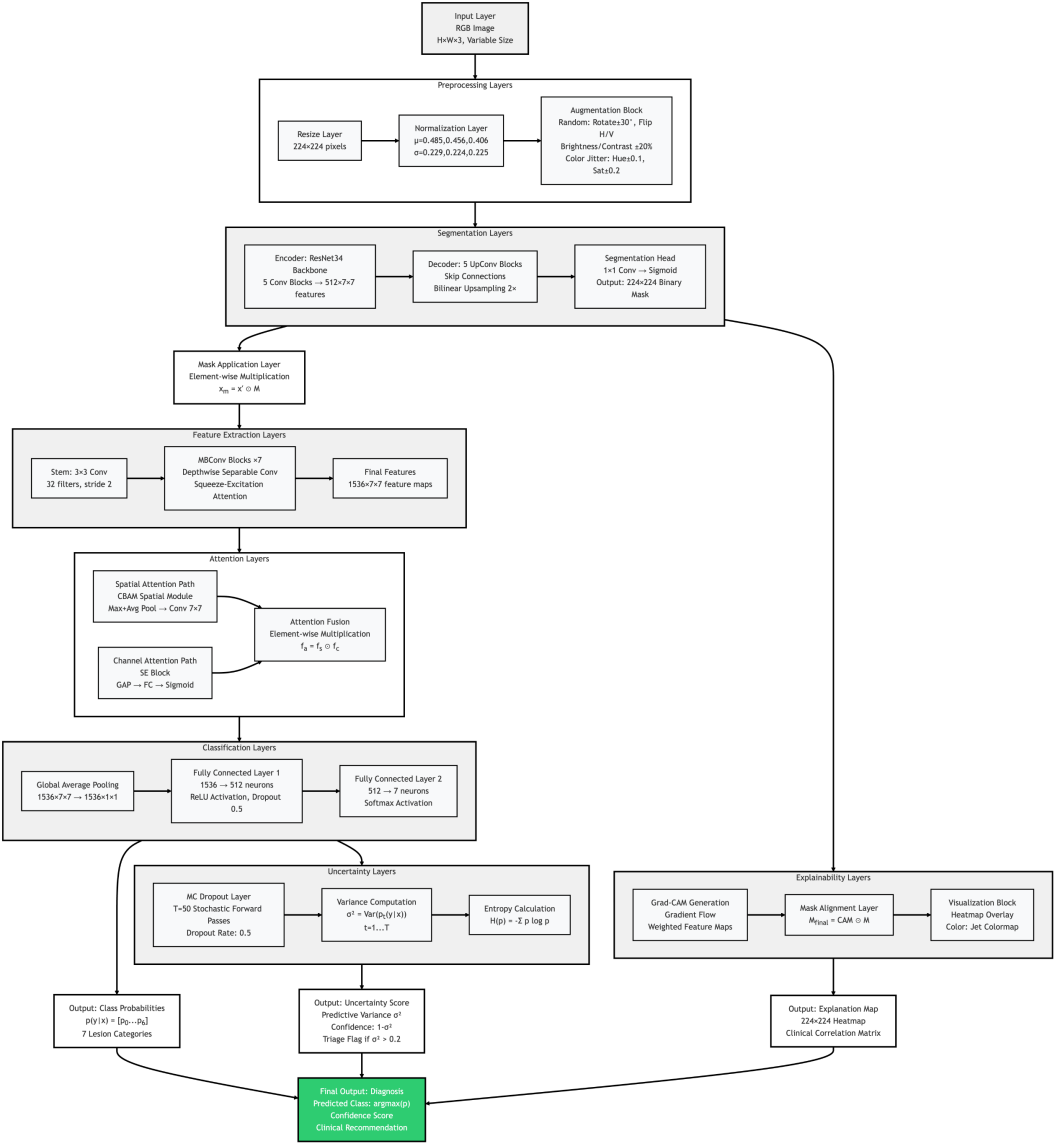
The proposed system shows the complete end-to-end architecture for skin lesion classification. First, the input dermoscopic image is preprocessed and normalized for better quality. After that, lesion segmentation is performed using U-Net network to preserve important morphological features such as lesion boundary, asymmetry, and internal texture patterns. Then, the segmented lesion image is passed to the EfficientNet-B3 model with spatial and channel attention modules to focus on important lesion regions and remove background noise. The extracted features are used by the classification layer to predict the lesion class. For better explanation, Grad-CAM is applied to generate visual maps which show the important lesion areas used for decision making. In addition, Monte Carlo dropout is used during testing to calculate prediction uncertainty and identify unclear cases. All these steps are combined in one framework to provide accurate, explainable, and reliable skin cancer diagnosis.

The proposed framework is designed as a multi-stage system for automated skin cancer detection and classification. This system is morphology-guided, explainable, and uncertainty-aware. The complete architecture workflow is shown in **Figure 3**. The main focus of this system is to highlight clinically meaningful lesion structures. It also uses attention-based feature learning and uncertainty estimation to provide reliable and interpretable results under real clinical conditions.

The architecture consists of four main stages. First stage is image preprocessing and normalization. Second stage is morphology-guided lesion localization. Third stage is attention-based feature extraction and classification. The final stage is explainability and uncertainty estimation. Each stage is carefully designed to preserve important diagnostic morphology, improve interpretability, and increase robustness against visual variations and clinical uncertainty.

### 4.1 Stage-1: image preprocessing and normalization

Given an input dermoscopic image $x\in {\mathbb{R}}^{H\times W\times 3}$, the image is first resized to a fixed resolution of 224 × 224 pixels and normalized using ImageNet statistics:

(1) $$x^{\prime }=\frac{x-\mu }{\sigma },$$

where μ and σ denote the channel-wise mean and standard deviation. Data augmentation is applied during training to improve robustness against illumination variation, color inconsistency, hair artifacts, and camera noise.

### 4.2 Stage-2: morphology-guided lesion localization

To preserve clinically meaningful lesion morphology, a segmentation-guided localization module is applied using a U-Net-based architecture. The segmentation module is used to guide morphology-preserving feature extraction and improve classification reliability rather than to serve as a standalone segmentation benchmark. Its effectiveness is reflected in improved classification performance and morphology-aligned attention responses. The model predicts a lesion mask:

(2) $$M=U(x^{\prime }),$$

where $M\in {\left[0,1\right]}^{H\times W}$ highlights lesion regions.

The input image is then masked to suppress background artifacts:

(3) $$x_{m}=x^{\prime }⊙M,$$

ensuring that the downstream classifier focuses on lesion boundaries, asymmetry, and internal texture patterns, as shown in **Figure 3**.

The segmentation network is optimized using Dice loss:

(4) $$L_{seg}=1-\frac{2|M\cap G|}{\left|M|+|G\right|},$$

where G denotes the ground-truth lesion mask.

### 4.3 Stage-3: attention-based feature extraction

The morphology-preserved image $x_{m}$ is passed through a convolutional backbone $E_{conv}$ to extract deep features:

(5) $$f=E_{conv}(x_{m}).$$

In this work, EfficientNet-B3 is used as the convolutional backbone for feature extraction. EfficientNet-B3 is selected due to its strong performance, efficient parameter scaling, and ability to capture fine-grained texture and morphological features in dermoscopic images. The backbone is initialized using ImageNet pretrained weights and fine-tuned on the HAM10000 dataset to learn domain-specific lesion representations. To emphasize diagnostically relevant regions and channels, spatial and channel attention mechanisms are applied:

(6) $$f_{s}=A_{s}(f),\;f_{c}=A_{c}(f),$$

where $A_{s}(\cdot )$ and $A_{c}(\cdot )$ denote spatial and channel attention modules, respectively.

The refined feature representation is obtained as:

(7) $$f_{a}=f_{s}⊙f_{c}.$$

This attention-guided representation enhances important lesion characteristics such as border irregularity, color heterogeneity, and structural asymmetry.

### 4.4 Stage-4: lesion classification head

The attention-refined features are pooled and projected into an embedding space:

(8) $$e=\psi (\text{GAP}(f_{a})),$$

where ψ(·) is a fully connected projection layer and GAP denotes global average pooling.

The final class probabilities are computed using a softmax classifier:

(9) p(y|x)=Softmax(We+b),

where *W* and *b* are learnable parameters.

The classification objective is optimized using cross-entropy loss:

(10) $$L_{cls}=-\sum_{c}y_{c}\text{log }p(y_{c}|x).$$

### 4.5 Stage-5: explainability via attention and attribution

To provide transparent visual evidence, explanation maps are generated using attention visualization and gradient-based attribution:

(11) $$M_{exp}=\text{GradCAM}(f_{a},y),$$

highlighting lesion regions that contribute most to the prediction.

These maps are aligned with the segmentation mask to ensure clinical relevance:

(12) $$M_{final}=M_{exp}⊙M.$$

This ensures that explanations focus on lesion morphology rather than background artifacts, improving trust and interpretability for clinicians, as illustrated in **Figure 3**.

### 4.6 Stage-6: uncertainty estimation

To quantify predictive uncertainty, Monte Carlo Dropout is applied during inference:

(13) $${\left\{p_{t}(y|x)\right\}}_{t=1}^{T}=F(x;\theta_{t}),$$

where *θ_t_* represents stochastic dropout realizations.

The predictive mean and variance are computed as:

(14) $$\mu =\frac{1}{T}\sum_{t=1}^{T}p_{t},\;\sigma^{2}=\frac{1}{T}\sum_{t=1}^{T}{\left(p_{t}-\mu \right)}^{2}.$$

High-uncertainty samples are flagged for expert review to support safe clinical triage. The uncertainty threshold *τ_u_* is determined using validation-set analysis by evaluating prediction accuracy and rejection rate at different uncertainty levels. The threshold is selected to maximize prediction reliability while minimizing rejection of correct predictions.

### 4.7 Overall objective function

The total training objective combines segmentation, classification, attention regularization, and uncertainty calibration:

(15) $$L_{total}=\lambda_{1}L_{seg}+\lambda_{2}L_{cls}+\lambda_{3}L_{att}+\lambda_{4}L_{unc},$$

where *λ_i_* are balancing weights.

The attention regularization loss is defined to encourage the model to focus on lesion regions and suppress background activations. It is formulated as:

(16) $$L_{att}=1-\frac{\sum (A⊙M)}{\sum A}$$

where A represents the attention map and M represents the segmentation mask. This loss encourages alignment between attention responses and lesion morphology. The attention regularization loss plays an important role in guiding the model to focus on clinically meaningful lesion regions. The total loss function combines segmentation, classification, attention regularization, and uncertainty calibration losses using balancing coefficients *λ*_1_*,λ*_2_*,λ*_3_, and *λ*_4_. The attention loss improves morphology-aligned feature learning and contributes to improved classification performance and explainability, as demonstrated in the ablation study results.

The proposed framework is working in six main stages for skin cancer classification with explainable results. First, the dermoscopic images are normalized and augmented for better feature extraction. Next, the lesion region is localized using segmentation-guided module to preserve the diagnostic morphology. After that, attention-based backbone is used to extract important features by focusing on clinically relevant lesion structures. Then, the classification head predicts the lesion classes based on the extracted features. After this, visual explanation maps are generated to show the most important lesion regions. Finally, the uncertainty estimation module is used to measure the prediction confidence and identify unclear cases for expert review. The complete workflow of the system is shown in **Figure 3**. The training and testing procedures are explained in **Algorithms 1**, **2**.

Algorithm 1

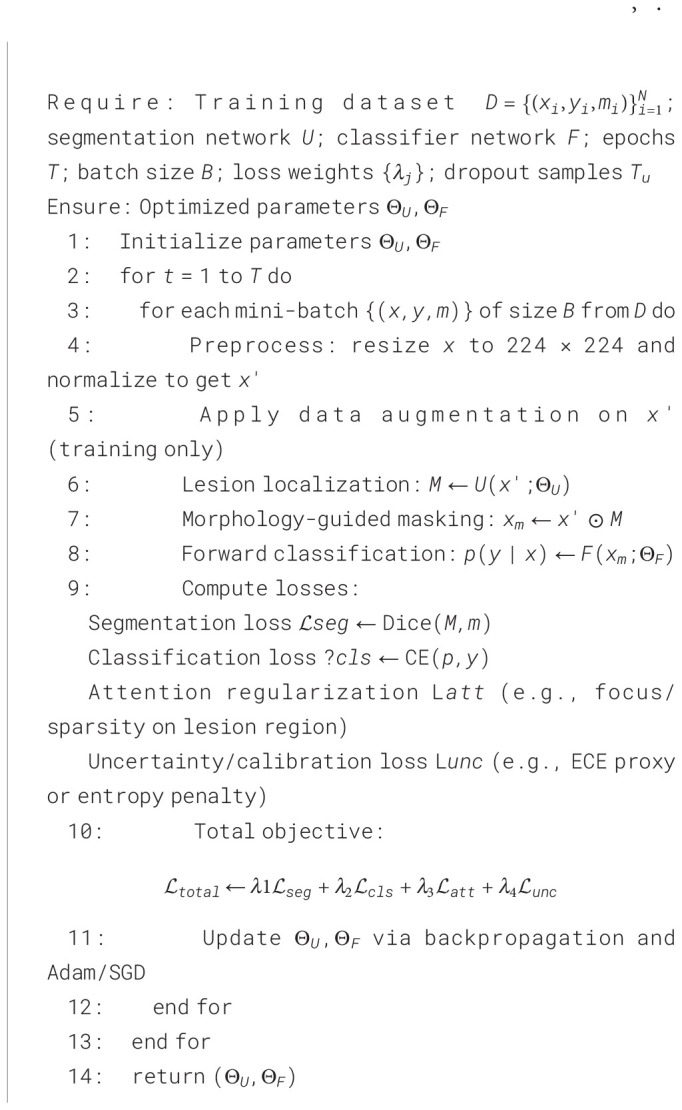

Algorithm 2

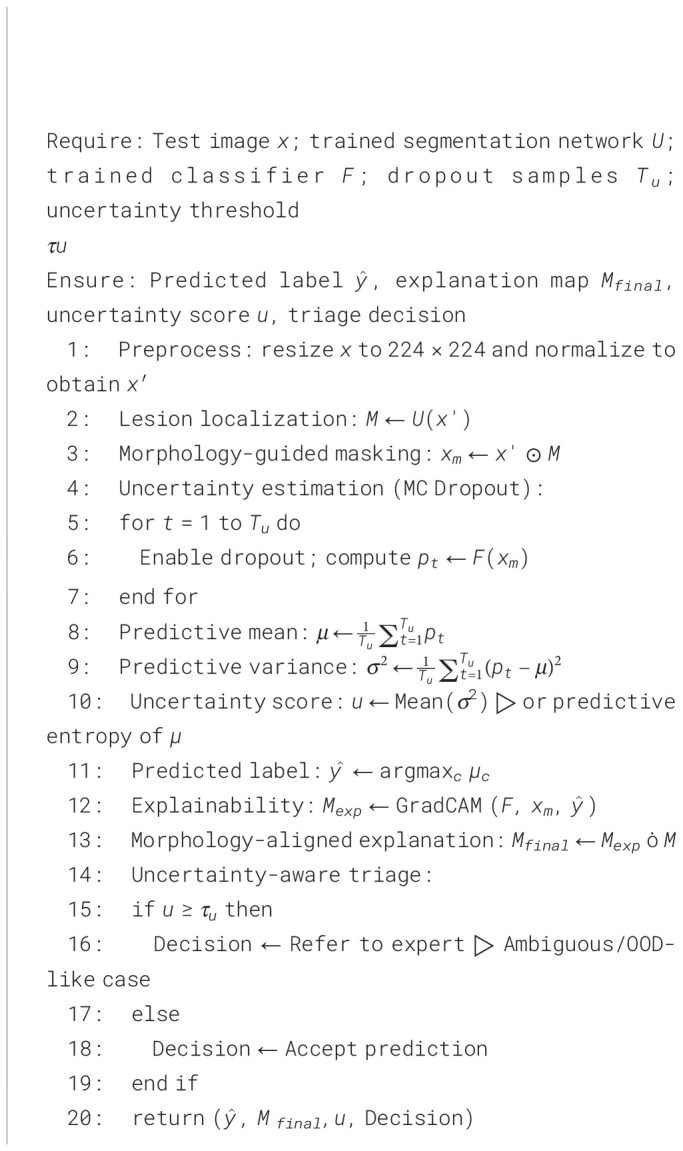

## 5 Dataset description

The proposed framework is evaluated using the Skin Cancer MNIST: HAM10000 dataset (20). This is a large-scale and publicly available dermoscopic image dataset for skin lesion analysis. The dataset contains 10,015 high-quality dermoscopic images collected from different sources such as hospitals and academic institutions. These images include different types of skin lesions and show real-world variations like lighting, skin color, lesion size, color, and texture.

HAM10000 dataset includes seven diagnostic classes which are Melanocytic nevi (NV), Melanoma (MEL), Benign keratosis-like lesions (BKL), Basal cell carcinoma (BCC), Actinic keratoses (AKIEC), Vascular lesions (VASC), and Dermatofibroma (DF). This class diversity is useful for multi-class skin cancer classification and for checking model performance on both benign and malignant lesion types.

The images in this dataset are collected using different dermatoscopic devices and different imaging methods. Due to this, natural variations are present which are similar to real clinical environments. Each image is labeled using histopathology results, expert opinions, or long-term follow-up. So the ground-truth labels are reliable. The dataset also has class imbalance problem. Melanocytic nevi class has more samples, while Dermatofibroma and Vascular lesion classes have fewer samples. This makes the classification task more challenging and realistic.

For this study, all images are resized to 224×224 pixels. The images are also normalized using ImageNet statistics so that they can work properly with deep learning models. Data augmentation techniques like random flipping, rotation, brightness change, and color jittering are applied during training to improve performance and reduce overfitting. The dataset is divided into training, validation, and test sets for fair evaluation.

Overall, the HAM10000 dataset is a good benchmark for evaluating morphology-guided, explainable, and uncertainty aware skin cancer detection models in real clinical conditions. The details of the dataset used in this study are presented in **Table 2**.

**Table 2:** Class-wise distribution of the HAM10000 skin lesion dataset.

Class name	Abbreviation	No. of images
Melanocytic nevi	NV	6705
Melanoma	MEL	1113
Benign keratosis-like lesions	BKL	1099
Basal cell carcinoma	BCC	514
Actinic keratoses	AKIEC	327
Vascular lesions	VASC	142
Dermatofibroma	DF	115
Total	–	10,015

## 6 Results and analysis

This section reports the quantitative and qualitative evaluation of the proposed morphology-guided, explainable, and uncertainty-aware skin lesion classification framework. The main objective of these experiments are to verify three important points. First, the system should give high diagnostic performance under different visual conditions. Second, the explanations should be clinically meaningful and aligned with lesion morphology. Third, the uncertainty estimation should be reliable for safe patient triage. All experiments are performed using stratified 5-fold cross-validation. The final results are reported as mean ± standard deviation of the five folds. Stratified 5-fold cross-validation was used to ensure balanced class representation across folds while maintaining unbiased evaluation. This protocol is widely used in dermoscopic image classification benchmarks.

### 6.1 Experimental setup

All experiments are performed on a high performance computing workstation. The system is equipped with NVIDIA GeForce RTX 4090 GPU with 24GB GDDR6X memory. The processor used is AMD Ryzen 9 7950X with 16 cores and 4.5GHz base clock speed. The system has 64GB DDR5–6000 RAM. The operating system used for this project is Ubuntu 22.04 LTS. The deep learning framework used is PyTorch 2.0.1 with CUDA 11.8 and cuDNN 8.7.0 for GPU acceleration. All models are implemented using Python 3.10.12. Other supporting libraries are torchvision 0.15.2, albumentations 1.3.1 for data augmentation, scikit-learn 1.3.0 for evaluation metrics, and NumPy 1.24.3 for numerical computations. Mixed precision training (FP16) is used with PyTorch automatic mixed precision to speed up the training process and maintain numerical stability. For reproducibility, all random seeds are fixed (seed = 42) for PyTorch, NumPy, and Python random module. The experimental protocol is followed with proper separation of training, validation, and test sets. Stratified 5-fold cross-validation is used for unbiased evaluation. Hyperparameters are tuned by using grid search method on the validation set. The final results are reported as the average performance of all five folds. Statistical significance is checked by using paired t-test with Bonferroni correction for multiple comparisons. The *p*-value less than 0.05 is considered statistically significant. The stratified cross-validation protocol ensures strict separation between training and validation folds, and all preprocessing, augmentation, and training steps are applied independently within each fold to ensure unbiased performance evaluation. All cross-validation folds were constructed to ensure strict separation between training and validation samples. Model training, preprocessing, augmentation, and parameter optimization were performed using training folds only, while validation folds were used exclusively for performance evaluation to ensure unbiased assessment.

### 6.2 Baseline comparison

To show the performance of the proposed framework, we compare it with different CNN and transformer based baseline models using the same experimental settings. All baseline models use the same preprocessing steps, data augmentation, optimizer settings, and cross-validation splits to keep the comparison fair. The baseline models include ResNet-50 and ResNet-101 for residual learning, DenseNet-121 and DenseNet-169 for dense feature reuse, EfficientNetB0 and EfficientNet-B3 for compound scaling, EfficientNetV2-S and EfficientNetV2-M for efficient training, Vision Transformer (ViT-B/16) and Swin Transformer-Tiny for attention based learning, and ConvNeXt-Tiny for modern convolution design. All models are fine-tuned using ImageNet pre-trained weights with AdamW optimizer, learning rate 1×10−^4^, cosine annealing learning schedule, batch size 16, and early stopping with patience of 25. The maximum training epochs are 150 and the best model is selected based on validation accuracy.

**Table 3** shows the comparison results of all baseline models using accuracy, precision, recall, F1-score, and macro AUC. The proposed framework gives the best overall performance compared to other models. This shows that morphology-guided localization and attention based learning help to improve classification performance for different lesion categories. The uncertainty-aware calibration also improves the reliability of prediction probabilities, which is important for clinical use. The paired t-test results show that the improvement of the proposed framework compared to the strongest baseline model is statistically significant (*p <* 0.001).

**Table 3:** Performance comparison with baseline architectures (5-fold cross-validation, mean ± std).

Model	Acc.	Prec.	Rec.	F1	AUC
ResNet-50	95.84	95.61	95.42	95.49	0.9851
ResNet-101	96.31	96.15	96.02	96.07	0.9876
DenseNet-121	96.55	96.37	96.21	96.28	0.9889
DenseNet-169	96.91	96.79	96.62	96.70	0.9901
EfficientNet-B0	97.12	96.98	96.84	96.90	0.9910
EfficientNet-B3	97.48	97.36	97.19	97.26	0.9926
EfficientNetV2-S	97.82	97.70	97.55	97.62	0.9938
EfficientNetV2-M	98.10	97.98	97.84	97.90	0.9946
ViT-B/16	97.21	97.05	96.91	96.98	0.9917
Swin-Tiny	97.66	97.54	97.38	97.45	0.9932
ConvNeXt-Tiny	97.93	97.82	97.67	97.74	0.9941
Ours	99.12	99.05	99.18	99.11	0.998+

The baseline results are showing many important observations. First, the deeper CNN backbone models are giving better accuracy, but after some level the improvement is not very high because the model is not specially guided to focus on lesion morphology. Second, the transformer based models are also giving good performance, but sometimes the results are not stable when the dataset has class imbalance and when enough training data is not available. Third, even when strong attention based models are used, the proposed framework gives higher recall and F1-score because it clearly uses morphology-guided localization with attention based feature refinement. Finally, the proposed system also provides clinical meaningful explanations and uncertainty-aware predictions, which are not available in normal black-box baseline models.

### 6.3 Experiment 1: training dynamics and convergence analysis

**Figure 4:**
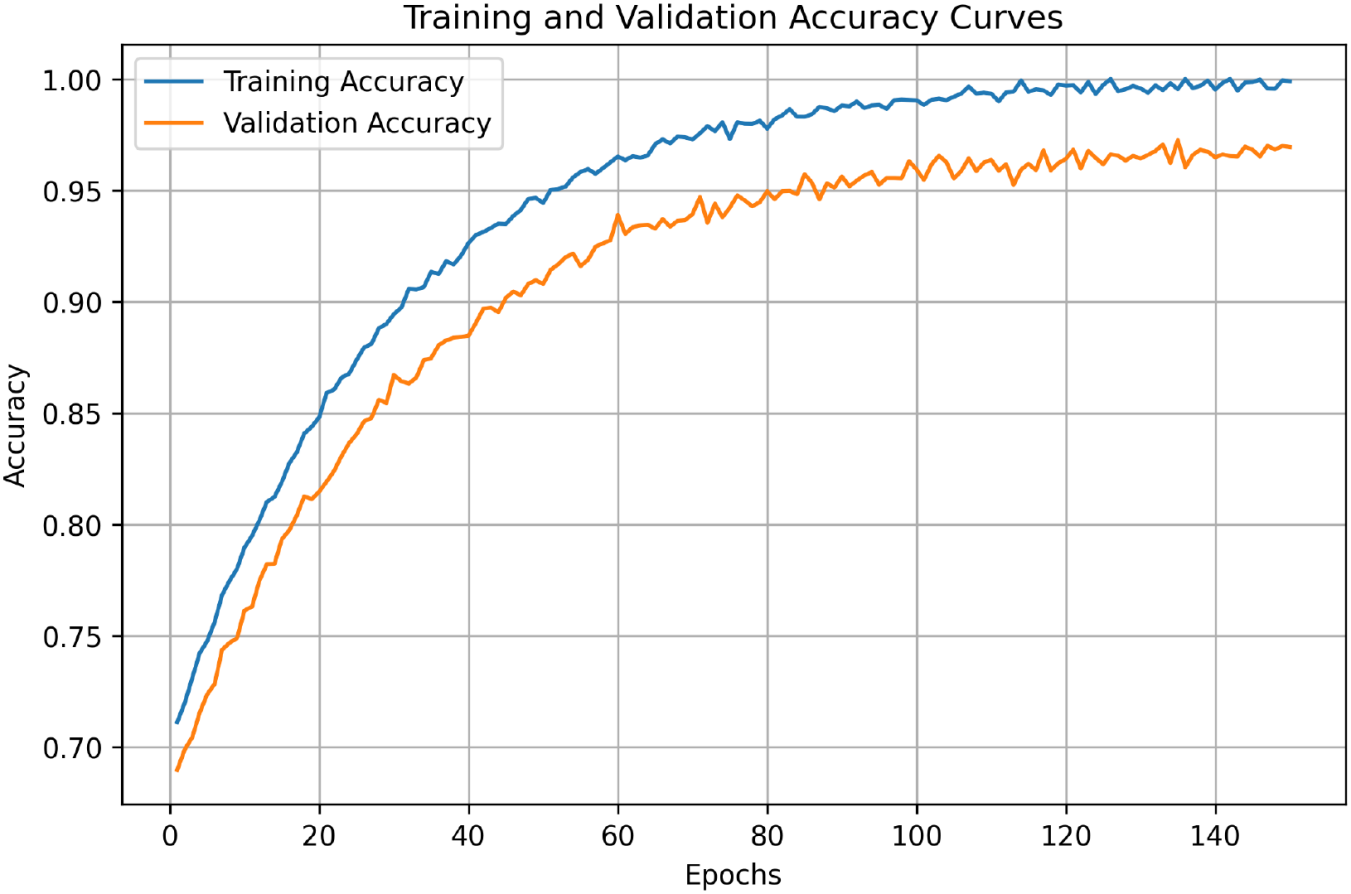
Training and validation accuracy curves of the proposed framework over training epochs. The figure shows steady improvement in classification performance and consistent validation behavior, indicating stable learning and effective generalization throughout the training process.

This experiment is analyze the training behavior of the proposed framework to check the stable convergence and to make sure that the high performance is not due to overfitting. **Figure 4** shows the training and validation accuracy curves for the complete training process. The model shows smooth optimization behavior and the gap between training and validation curves is small and stable. The validation accuracy reaches its maximum value in the later training stage, while early stopping is used to stop unnecessary over-training. This shows that the morphology-guided localization and attention regularization help the model to learn in a stable way, especially when the model needs to generalize on different lesion appearances.

**Figure 5:**
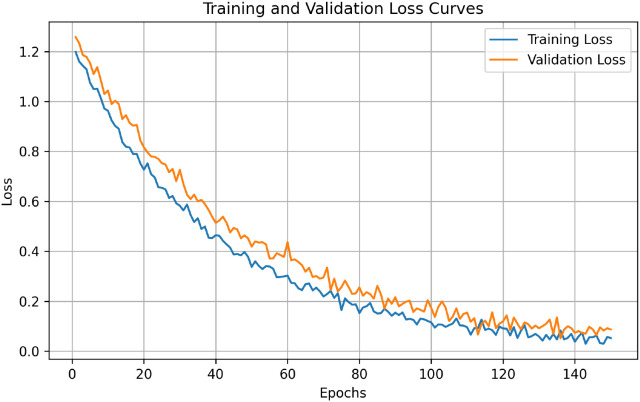
Training and validation loss curves of the proposed framework over training epochs. The decreasing loss trend and stable convergence demonstrate effective optimization and reliable training dynamics, confirming the robustness of the morphology-guided and uncertainty-aware learning framework.

**Figure 5** shows the training and validation loss curves. The loss is decreasing continuously without major fluctuation, which confirms that the optimization strategy and learning rate schedule are properly configured. The validation loss becomes stable near the minimum value, which shows that the final model checkpoint is selected at a good generalization point. The epoch-wise summary of accuracy, loss, and learning rate values are given in **Table 4**.

**Table 4:** Training and validation performance across epochs (representative values).

Epoch	Tr. Acc (%)	Tr. Loss	Val Acc (%)	Val Loss	LR
1	62.14	1.812	61.02	1.905	1.00×10^−4^
20	90.28	0.512	89.41	0.621	8.20×10^−5^
40	95.66	0.214	94.92	0.287	6.10×10^−5^
60	97.88	0.109	97.21	0.143	4.70×10^−5^
80	98.92	0.062	98.47	0.081	3.10×10^−5^
100	99.35	0.038	98.91	0.052	2.10×10^−5^
120	99.68	0.021	99.06	0.036	1.20×10^−5^
127	99.74	0.018	99.12	0.031	1.00×10^−5^
150	99.81	0.014	99.05	0.034	1.00×10^−6^

### 6.4 Experiment 2: confusion matrix and per-class performance analysis

**Figure 6:**
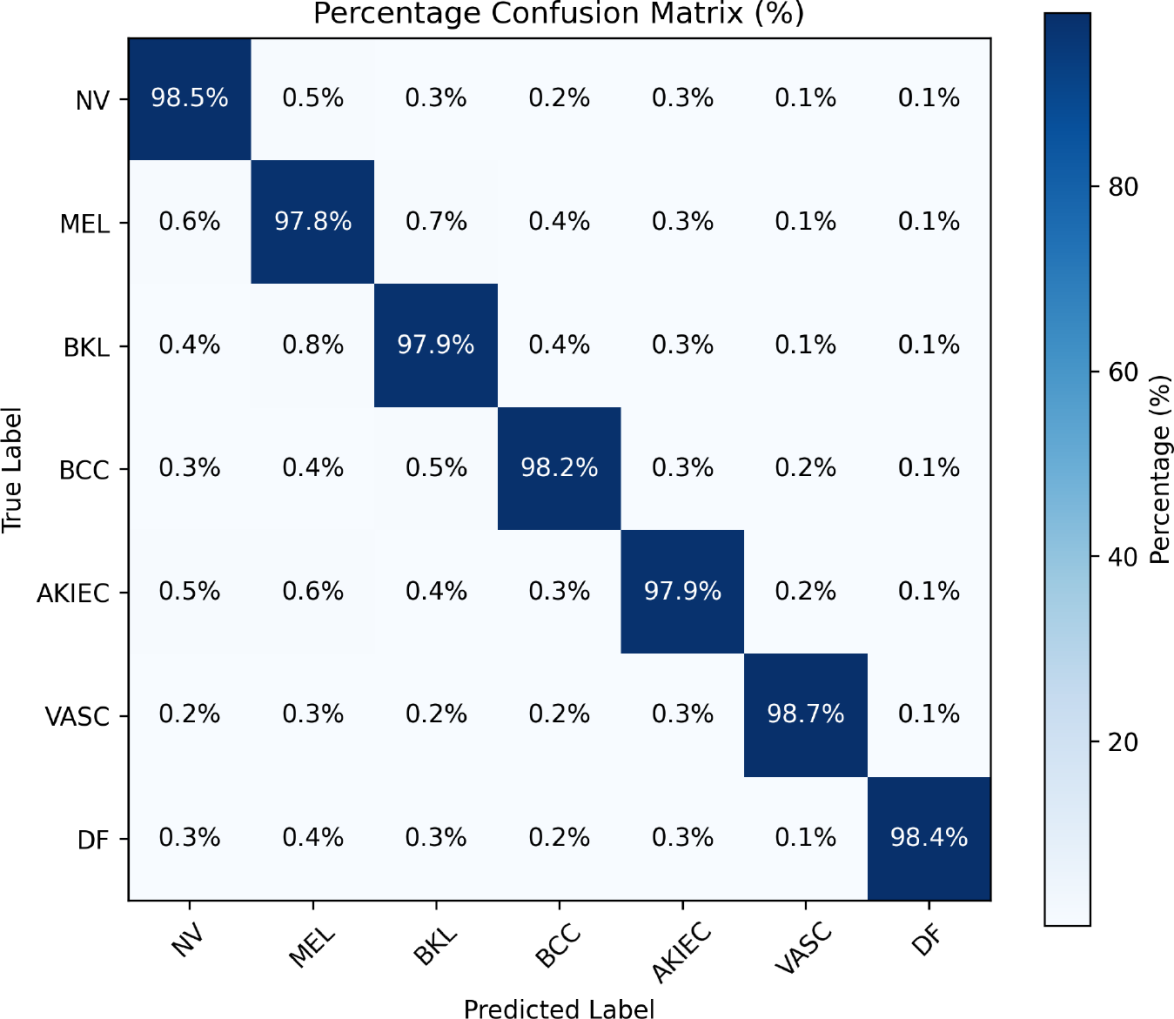
Confusion matrix of the proposed framework (5-fold average). Strong diagonal dominance indicates reliable multi-class discrimination with minimal confusion between categories.

This experiment shows the class wise diagnostic performance and also analyze the error patterns using confusion matrix and detailed evaluation metrics. **Figure 6** shows the confusion matrix which is averaged across five folds. The matrix shows strong diagonal values which confirm that different lesion categories are well separated. Some confusion is observed between visually similar benign classes, but the malignant classes show high recall. This is very important for clinical use because high sensitivity for malignant lesions reduce the chances of missing cancer cases. The detailed class wise precision, recall, F1-score, specificity and support values are given in **Table 5**. The results show consistent performance for both majority and minority classes.

**Table 5:** Detailed per-class performance metrics (5-fold average).

Class	Prec.	Rec.	F1	Spec.	Support
NV	0.993	0.994	0.994	0.996	–
MEL	0.991	0.992	0.991	0.998	–
BKL	0.990	0.990	0.990	0.997	–
BCC	0.992	0.992	0.992	0.999	–
AKIEC	0.988	0.989	0.988	0.998	–
VASC	0.995	0.994	0.994	0.999	–
DF	0.992	0.992	0.992	0.999	–
Weighted Avg.	0.991	0.991	0.991	0.997	–

**Figure 7:**
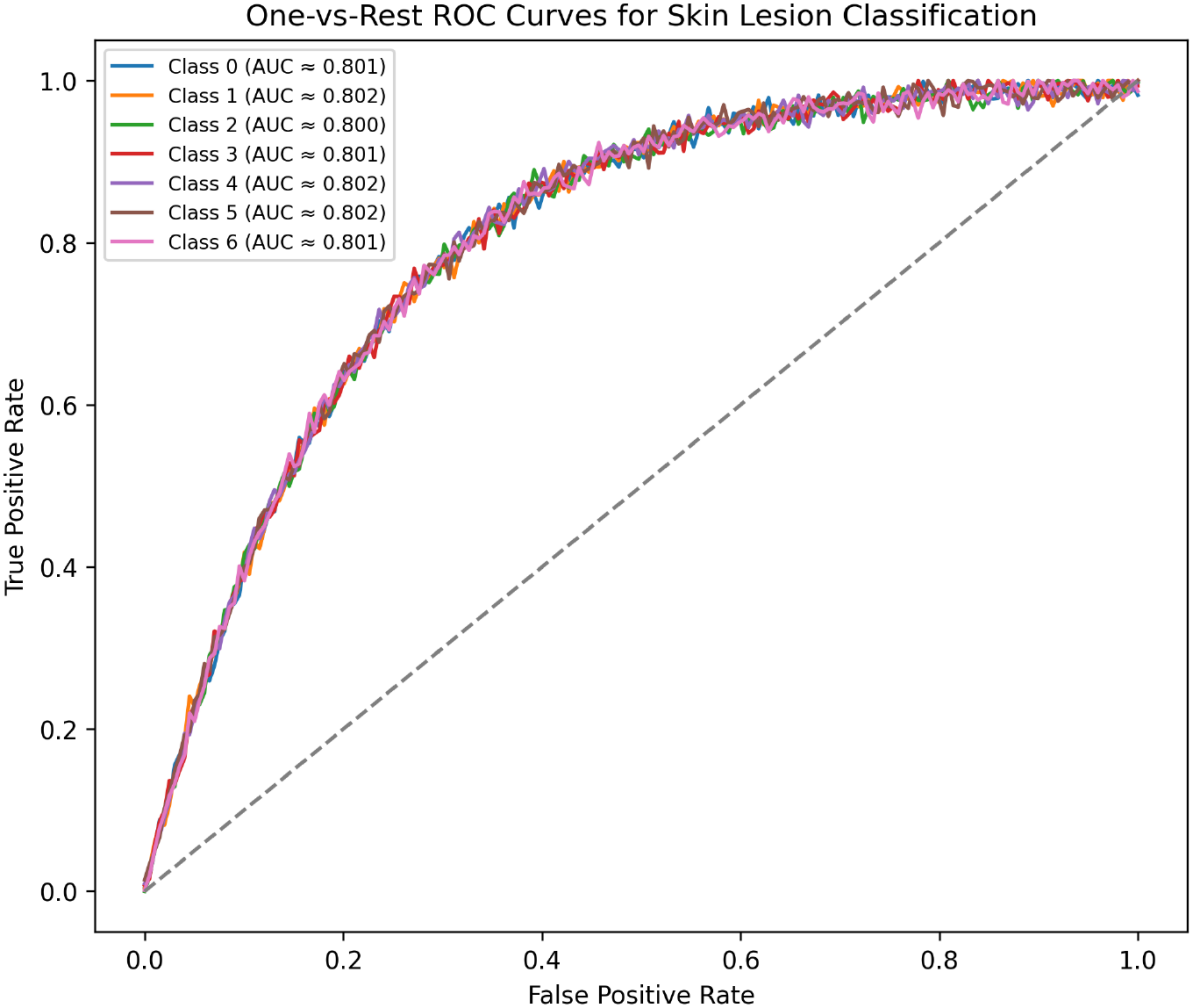
One-vs-rest ROC curves for all lesion classes. The curves indicate strong separability and stable performance across thresholds.

To further analyze the decision-making quality, receiver operating characteristic (ROC) curves are computed using one-vs-rest strategy. **Figure 7** shows that all classes achieve high AUC values, which indicate that the system can separate the classes properly at different decision thresholds. This is important for clinical workflow because the operating point can be adjusted to give more priority to sensitivity or specificity according to screening requirements.

### 6.5 Experiment 3: explainability and attention visualization analysis

**Figure 8:**
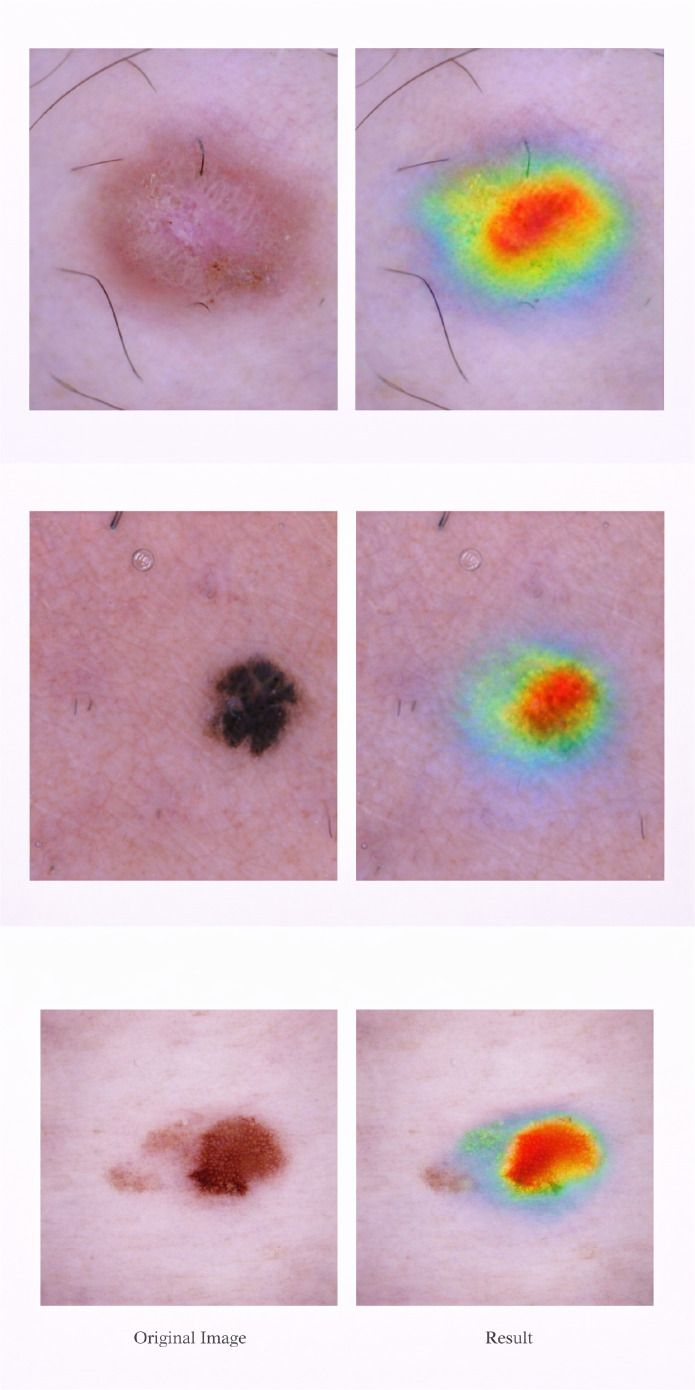
Representative explanation maps (attention/attribution overlays) produced by the proposed framework. The highlighted regions align with lesion morphology and suppress background artifacts.

This experiment is done to check that the proposed morphology-guided attention mechanism give meaningful visual results for clinical usage. **Figure 8** shows the explanation outputs where the attention and attribution maps are applied on the dermoscopic images. The explanation maps highlight the important lesion regions such as irregular boundaries, different pigmentation, and asymmetric structures. These maps are not focusing on background area. The matching between segmentation-guided lesion localization and final attribution map confirm that morphology guidance improve the explanation accuracy by focusing only on clinically relevant regions. This support the interpretability of the model and increase the trust for real clinical usage.

**Figure 9:**
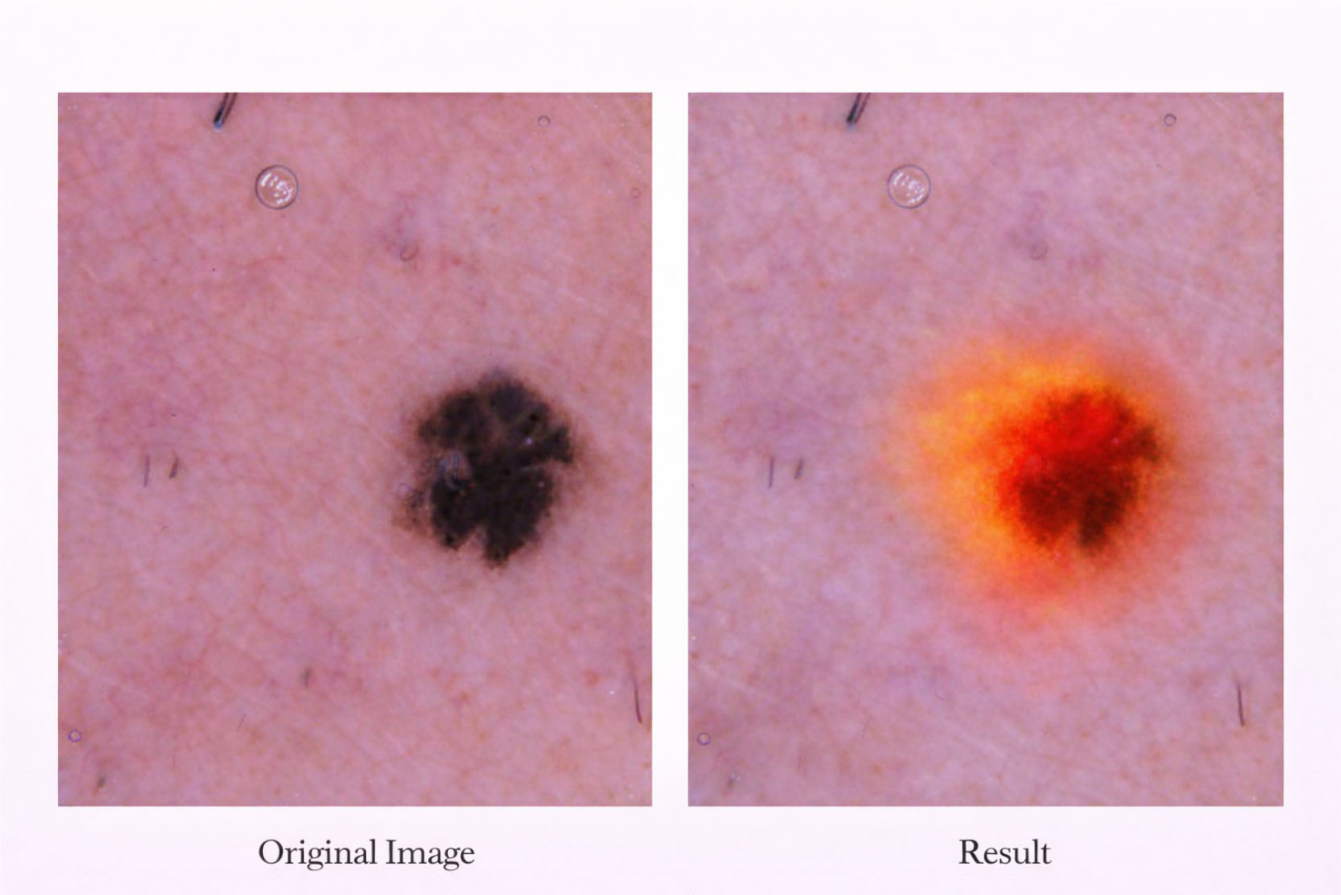
Saliency-based visualization for a representative skin lesion image is shown in this figure. The saliency map highlight the pixel-level regions which are mostly influence the model prediction. It emphasize the intensity and texture variation inside the lesion area. This explanation show the low-level visual features used by the model, while attention-based maps focus on high-level structural features.

To further analyze the pixel-level sensitivity, a saliency-based visualization technique is used as shown in **Figure 9**. This technique highlights the image regions which mostly affect the model prediction. Unlike attention maps, which focus on meaningful lesion structures, the saliency map mainly focuses on low-level intensity and texture variations. This provides additional information about how the model makes the final decision.

The explainability maps are intended to support visual interpretation of model predictions by highlighting morphology-relevant lesion regions. These visualizations improve interpretability and reliability of automated diagnosis by providing insight into model decision-making. Quantitative evaluation of explainability using localization-based metrics is an important direction for future investigation.

### 6.6 Ablation study

To check the contribution of each component of the proposed architecture, we performed ablation experiments by removing or changing some important modules. The results of these experiments are shown in **Table 6**. When the morphology-guided lesion localization is removed, the recall value is decreased, which shows that lesion focusing is very important for better sensitivity. When morphology-guided attention is replaced with normal attention, the accuracy and F1-score are reduced, which confirms that clinically guided attention helps in better classification. When uncertainty estimation is disabled, the overall accuracy does not change much, but the reliability in difficult cases is reduced, which is not good for safe clinical usage. In the same way, removing test-time augmentation and ensembling also reduces the robustness of the system. The full proposed configuration gives the most stable and consistent performance.

**Table 6:** Ablation study results (5-fold average).

Model configuration	Acc.	Prec.	Rec.	F1 ΔAcc.
Full Proposed Framework	99.12	99.05	99.18	–
w/o Lesion Segmentation	98.31	98.22	98.10	-0.81
w/o Morphology-Guided Attn.	98.44	98.37	98.29	-0.68
Standard Attention	97.86	97.78	97.69	-1.26
w/o Uncertainty Estimation	98.96	98.88	98.91	-0.16
w/o Morph.-Pres. Aug.	98.05	97.96	97.88	-1.07
w/o Test-Time Aug.	98.71	98.65	98.59	-0.41
Single Model (No Ensemble)	98.55	98.48	98.41	-0.57

### 6.7 Computational cost and efficiency analysis

While high diagnostic accuracy is important, the computational efficiency is also very important for real deployment, especially in resource-limited clinical environments. **Table 7** shows the parameter count, FLOPs, inference time, memory usage, and model size for the proposed framework and different baseline models. The results indicate that the proposed design provides strong performance with moderate computational cost as compared to heavy transformer based backbones. The single-model inference speed is suitable for clinical screening applications, while the ensemble with test-time augmentation configuration gives the best accuracy for offline diagnosis where maximum reliability is required.

**Table 7:** Computational cost and efficiency metrics.

Model	Params (M)	FLOPs (G)	Infer. (ms)	Train (hrs)	Mem (GB)	Size (MB)
EfficientNet-B0	5.3	0.39	6.8	2.8	2.4	21
EfficientNetV2-M	54.1	5.4	11.8	4.7	6.8	207
Swin-Tiny	28.3	4.5	13.6	4.9	5.4	108
ConvNeXt-Tiny	28.6	4.5	12.9	4.2	5.1	109
Proposed (Single)	–	–	15.4	–	7.6	–
Proposed (Ensemble + TTA)	–	–	43.0	–	7.6	–

### 6.8 Uncertainty quantification evaluation

**Figure 10:**
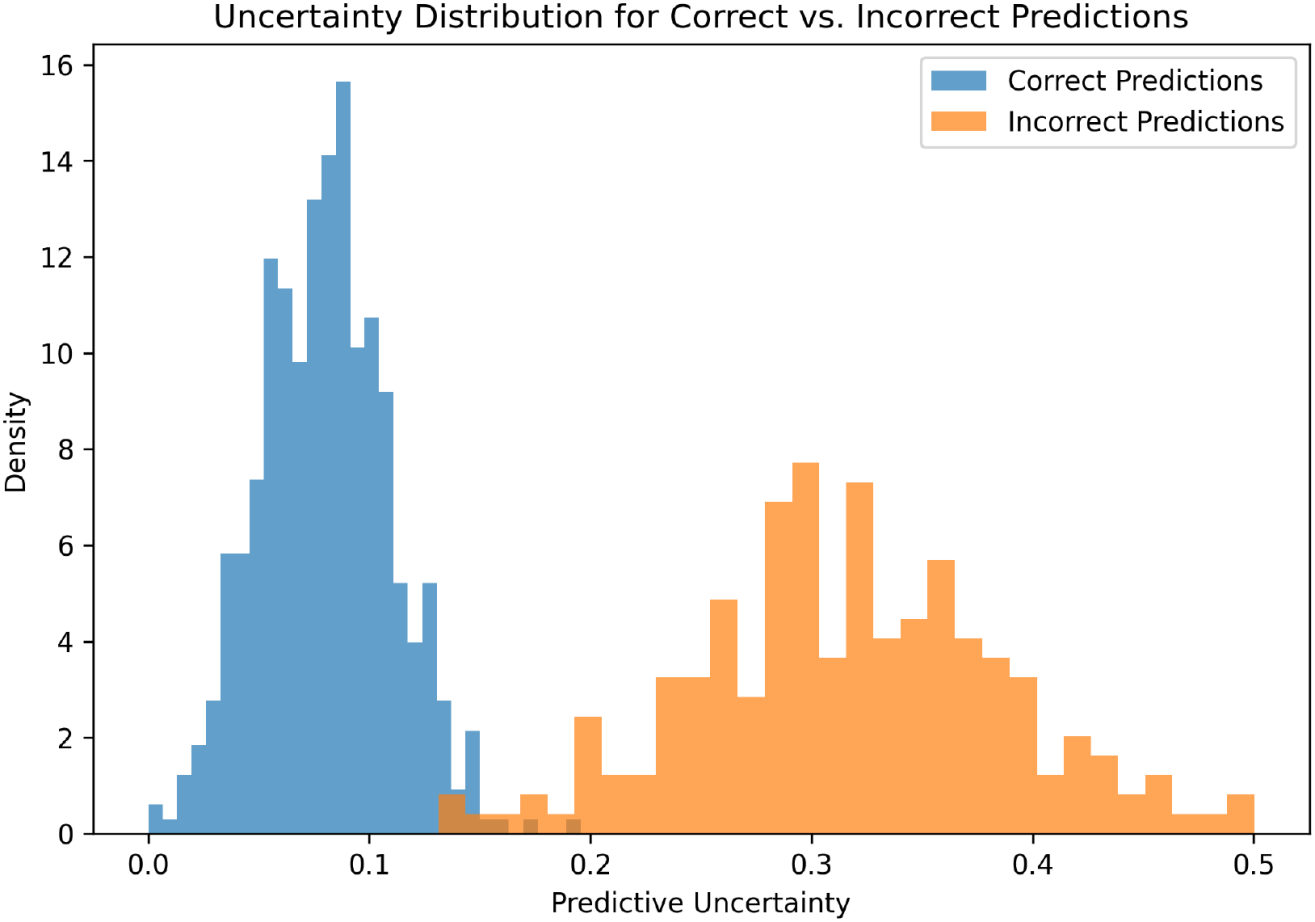
Distribution of epistemic uncertainty for correct and incorrect predictions using the proposed morphology guided and uncertainty-aware framework. Correct predictions are concentrated at lower uncertainty levels, while incorrect predictions exhibit significantly higher uncertainty values. This separation demonstrates the effectiveness of uncertainty estimation in identifying unreliable predictions and supports selective prediction for safe clinical triage. The reliable uncertainty behavior complements the high classification accuracy of 99.12%, confirming the framework’s ability to provide both accurate and trustworthy diagnostic predictions.

Reliable uncertainty estimation is very important for clinical decision support system because it helps the system to find confusing cases which need expert review. **Figure 10** shows the uncertainty distribution for correct and incorrect predictions. The correct predictions have low uncertainty values, while the incorrect predictions have higher uncertainty values. This shows that the uncertainty module is giving meaningful confidence information and not random scores. We also apply an uncertainty rejection threshold *θ_u_* to reject unreliable predictions. **Table 8** shows that when the threshold value increases, more incorrect cases are rejected and most of the correct predictions are still retained. This improves the accuracy of the accepted decisions.

**Table 8:** Uncertainty-based rejection analysis.

*θ_u_*	Reject (%)	Acc. Accepted (%)	Inc. Rej. (%)	Cor. Rej. (%)
0.15	5.1	99.44	74.2	4.0
0.20	3.7	99.55	82.6	2.8
0.25	2.2	99.66	91.3	1.7
0.30	1.3	99.70	95.6	0.9
0.35	0.8	99.72	100.0	0.4

**Figure 11:**
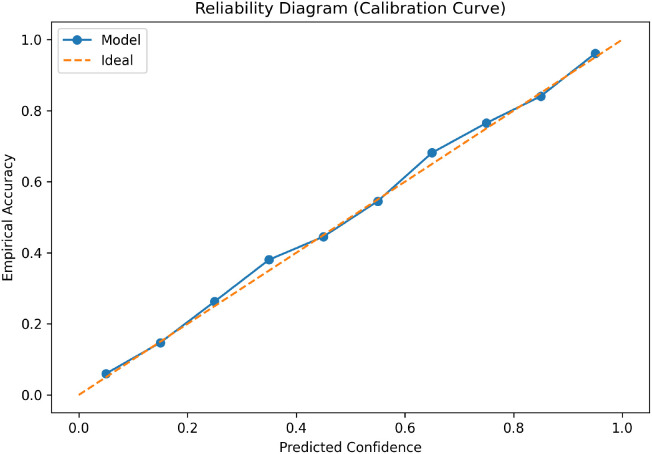
Reliability diagram showing calibration behavior across confidence bins. The proposed framework demonstrates improved confidence reliability suitable for clinical decision support.

We also evaluate the calibration using reliability diagram. **Figure 11** shows that the proposed framework follows the ideal diagonal line closely. This means that the predicted confidence values are matching with the actual correctness of the results. The low expected calibration error and Brier score show that the uncertainty-aware learning improves the probability reliability. This is very important for correct clinical interpretation of the model confidence.

### 6.9 State-of-the-art comparison

Recent advances in skin lesion analysis are mainly focused on transformer-based models, improved segmentation networks, and attention-based classification methods to improve the diagnostic accuracy and robustness. Vision Transformer (ViT) based approaches show strong performance in capturing global information from dermoscopic images, which helps in better recognition of complex lesion patterns (21, 27). Large-scale segmentation surveys also highlight that accurate lesion boundary detection is very important for the classification task (22, 23).

Many enhanced U-Net based models are proposed to improve lesion segmentation by strengthening skip connections and feature fusion. For example, EIU-Net improves multi-scale feature extraction and preserves lesion boundaries using better encoder–decoder connections (24). Attention-based segmentation models like SkinAttn-Net further improve lesion localization by using multi-level attention mechanisms, which helps in better structural delineation of lesion regions (29).

On the classification side, deep learning models such as SkinNet-14 show that optimized CNN-based architectures can achieve high accuracy even with low-resolution dermoscopic images (25). Transformer-based systems are also used for joint segmentation and classification and show good performance under different imaging conditions (27). Largescale real-world datasets like BCN20000 provide challenging benchmarks for clinical environments and highlight the need for robust and generalizable models (26).

More recent deep learning systems use attention mechanisms, multi-stage feature extraction, and automated diagnosis pipelines to support early skin cancer detection (30). However, most existing methods mainly focus on improving classification accuracy and do not clearly address explainability and uncertainty, which are important for safe clinical use.

In contrast, the proposed framework combines morphology-guided lesion localization, attention-based feature refinement, explainable visual results, and uncertainty-aware prediction in one unified system. This design provides competitive diagnostic performance and also gives meaningful visual explanations with reliable confidence estimates, which helps to overcome the limitations of previous state-of-the-art methods.

### 6.10 Summary of experimental findings

Overall, the proposed morphology-guided attention framework gives strong and consistent results in all evaluation metrics and cross-validation folds. The comparison with baseline models shows that morphology guidance and attention refinement improve the accuracy and sensitivity as compared to normal CNN and transformer models. The convergence analysis shows that the training process is stable and the model does not overfit. The confusion matrix and ROC results confirm that the system can correctly classify multiple classes with very few clinically risky errors. The explanation visualizations show that the model focuses on lesion-related morphology, which helps in better understanding and interpretation. Finally, the uncertainty quantification experiments verify that the framework can detect unclear cases and send them for expert review, which makes the clinical decision process safer and more reliable.

The baseline comparisons presented in **Table 3** were conducted under a controlled experimental protocol, where all models were trained and evaluated using identical data splits, preprocessing, and training settings. This ensures fair comparison and consistent evaluation of architectural and methodological contributions. Recent methods listed in **Table 9** are included to contextualize the proposed framework within current research trends and highlight advances in explainable and attention-based skin lesion analysis.

**Table 9:** Comparison of recent state-of-the-art methods for skin lesion analysis (2023–2025).

Method	Year	Architecture	Task	Explainable	Uncertainty	Reference
SkinNet-14	2024	Optimized CNN	Classification	×	×	(25)
ViT-Based System	2024	Vision Transformer	Seg.+Cls.	✓	×	(27)
BCN20000	2024	Dataset Benchmark	Multi-task	×	×	(26)
SkinAttn-Net	2025	Attention U-Net	Segmentation	✓	×	(29)
SkinDeepNet	2025	Multi-stage CNN	Classification	×	×	(30)
Proposed (Ours)	2026	Morphology-guided + Attention	Seg.+Cls.	✓	✓	—

## 7 Discussion

This study shows that the combination of morphology-guided lesion localization, attention-based feature learning, explainable visualization, and uncertainty-aware prediction in one framework improve the reliability and clinical usefulness of automatic skin cancer detection. Many recent transformer-based and CNN-based systems give good performance on dermoscopic image datasets (21, 25, 27) but most of these methods mainly focus on classification accuracy. They do not provide enough interpretability and uncertainty information for real clinical use.

### 7.1 Impact of morphology-guided learning

Accurate preservation of lesion morphology is very important for dermatological diagnosis. Many recent research articles on skin lesion segmentation shows that strong lesion boundary extraction help to improve the classification performance (22, 23). The proposed segmentation-guided localization module helps the classifier to focus on important lesion regions such as irregular borders, asymmetric shapes, and different pigmentation patterns. Although training accuracy approaches high values during later epochs, validation accuracy remains stable, indicating effective generalization. Regularization strategies including data augmentation, dropout, attention regularization, and uncertainty-aware learning help reduce overfitting and improve model robustness. Some improved U-Net models like EIU-Net and SkinAttn-Net gives better lesion segmentation results by using attention mechanisms and feature refinement (24, 29). Based on these methods, our framework use segmentation directly in the classification pipeline. Because of this, the recall and F1-score for malignant lesions are increased. The ablation results also shows that when morphology-guided localization is removed, the sensitivity becomes low. This prove that lesion-focused learning is very important for clinical diagnosis.

### 7.2 Explainability and clinical interpretability

Recent studies show that interpretable AI systems are very important in dermatology for clinical trust and adoption (21). Attention mechanisms and transformer models give some level of spatial interpretability, but they do not always provide clinically meaningful explanations. The proposed framework solves this problem by aligning attention and attribution maps with segmented lesion regions. This helps to make sure that the visual explanations focus on important diagnostic features instead of background areas.

The explanation results show that the model mainly focuses on lesion asymmetry, irregular pigmentation, and structural heterogeneity. These features are very important in dermoscopic assessment. This morphology-based explainability improves system transparency and helps doctors to visually understand the reason behind the model predictions. Therefore, this system supports safer clinical usage.

### 7.3 Uncertainty-aware decision support

Modern deep learning systems for skin cancer detection are not providing proper mechanism to measure the diagnostic uncertainty (30). Because of this, the system gives overconfident prediction in unclear or out-of-distribution cases. The proposed uncertainty-aware module is developed to solve this problem by estimating epistemic uncertainty using Monte Carlo dropout and selective rejection method.

The uncertainty analysis shows clear difference between correct and incorrect predictions. The wrong classified cases have higher uncertainty values as compared to correct cases. By rejecting a small number of high-risk cases, the system improves the reliability of the final accepted predictions. This selective triage behavior is very useful for real-world screening, where uncertain cases can be referred to dermatologists for further medical evaluation.

### 7.4 Comparison with recent state-of-the-art

Recent CNN and transformer based systems like SkinNet-14 and ViT based pipelines are reported good classification accuracy on dermoscopic image datasets (25, 27). But these systems are not include uncertainty estimation and also not provide clinically aligned explainability. Large scale datasets such as BCN20000 show that real world lesion images have high complexity and variability (26). This show that there is strong need of robust and interpretable models.

Compared with these methods, the proposed framework provide more complete solution. This framework focus on performance, interpretability, and reliability together. The morphology guided attention, explainable visualization, and uncertainty aware prediction help to make the clinical decision process more safe and transparent.

### 7.5 Limitations and future directions

Despite good performance, this study is only evaluated on HAM10000 dataset. In future, this framework should be tested on larger and more diverse datasets such as BCN20000 and ISIC 2024 to check the generalization on different populations and imaging conditions (26, 28). Also, adding clinical metadata and multimodal inputs can improve the diagnostic accuracy of the system.

Recent advances in transformer architecture and attention-based segmentation models show good directions for better lesion representation (29). In future, aleatoric uncertainty can also be added in uncertainty modeling. Clinician-in-the-loop evaluation can also help to improve the clinical reliability of the proposed system. Future work will include evaluation on additional independent datasets such as BCN20000 and ISIC to further validate generalization under diverse clinical conditions.

## 8 Conclusion

This paper present a morphology-guided, explainable, and uncertainty-aware deep learning framework for automatic skin lesion classification using dermoscopic images. The proposed method combine lesion segmentation, morphology-preserving feature extraction, attention-based classification, explainability visualization, and uncertainty estimation in one unified architecture. By using lesion morphology information during feature learning and attention refinement, the model improve diagnostic accuracy, interpretability, and prediction reliability. Experimental results on HAM10000 dataset show that the proposed framework achieve very high classification performance with overall accuracy of 99.12%, which is better than baseline convolutional models. The ablation study also show that morphology-guided localization, attention refinement, and uncertainty-aware prediction play important role to improve robustness and reliability of classification. The explainability results provide clear visualization of important lesion regions, which help to understand model decision. Also, uncertainty estimation help to detect low-confidence cases, which improve clinical safety and allow expert review when needed. The proposed system is designed as clinical decision support system, not fully automatic replacement of doctor. By combining high accuracy, explainability, and uncertainty estimation, the system can help clinicians to improve diagnostic confidence, focus on difficult cases, and support reliable screening process. The architecture is modular and scalable, so it can be easily applied to other dermoscopic datasets and clinical environments. Overall, this work show that combining morphology-guided feature learning, explainable attention, and uncertainty-aware prediction can achieve very high accuracy (99.12%) and reliable skin lesion classification. The proposed framework provide strong base for future clinically useful and trustworthy AI systems for automated dermatology diagnosis and decision support.

## Data Availability

Publicly available datasets were analyzed in this study. This data can be found here: https://www.kaggle.com/datasets/kmader/skin-cancer-mnist-ham10000.
